# Current evidence of climate‐driven colour change in insects and its impact on sexual signals

**DOI:** 10.1002/ece3.11623

**Published:** 2024-07-02

**Authors:** Md Tangigul Haque, Md Kawsar Khan, Marie E. Herberstein

**Affiliations:** ^1^ School of Natural Sciences Macquarie University Sydney New South Wales Australia; ^2^ Department of Biology, Chemistry and Pharmacy Free University Berlin Berlin Germany

**Keywords:** climate change, colour, insects, sexual selection, sexual signals

## Abstract

The colours of insects function in intraspecific communication such as sexual signalling, interspecific communication such as protection from predators, and in physiological processes, such as thermoregulation. The expression of melanin‐based colours is temperature‐dependent and thus likely to be impacted by a changing climate. However, it is unclear how climate change drives changes in body and wing colour may impact insect physiology and their interactions with conspecifics (e.g. mates) or heterospecific (e.g. predators or prey). The aim of this review is to synthesise the current knowledge of the consequences of climate‐driven colour change on insects. Here, we discuss the environmental factors that affect insect colours, and then we outline the adaptive mechanisms in terms of phenotypic plasticity and microevolutionary response. Throughout we discuss the impact of climate‐related colour change on insect physiology, and interactions with con‐and‐heterospecifics.

## Graphical abstract

Our review will provide current information about how climatic factors, such as temperature, precipitation, and humidity impact on insect's colour and sexual signals. In addition, we discussed about the counter mechanisms that insects usually take place to adapt with the changing climate. Finally, we identified current research gaps and highlighted potential future research areas.

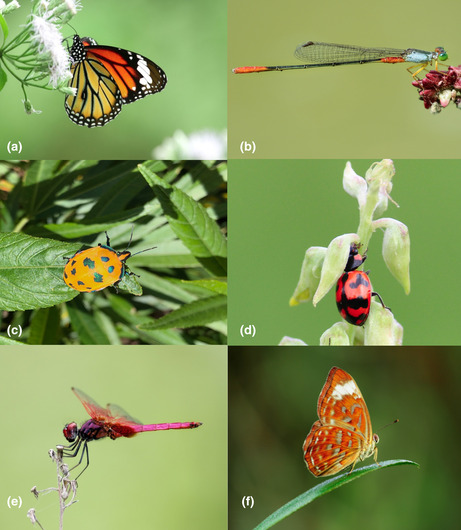

## 1 INTRODUCTION

**FIGURE 1:**
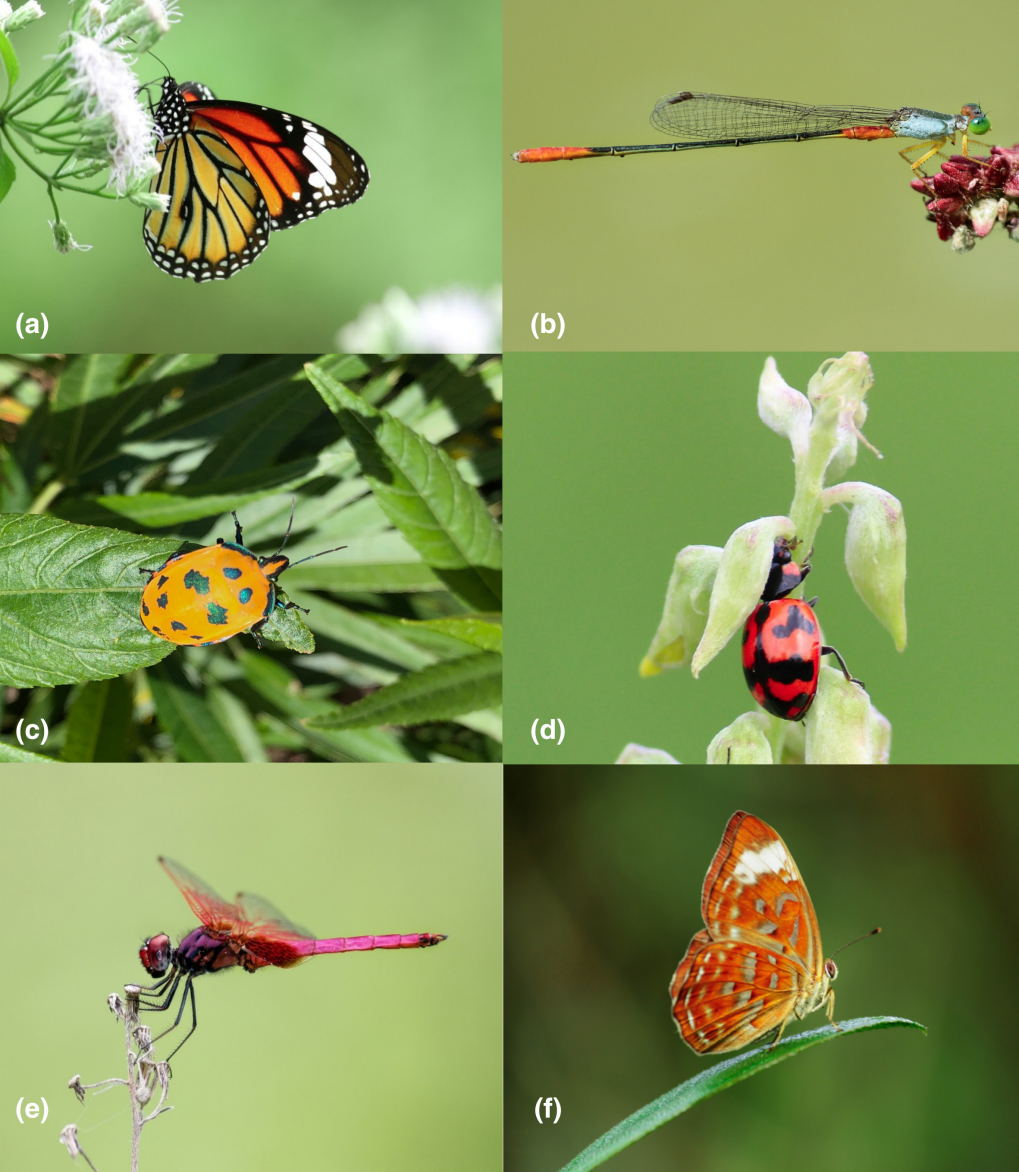
Insects exhibit diverse colours that are produced from pigments, structural‐based colour or a combination of both. (a) *Danaus genetia*, (b) *Ceriagrion cerinorubellum*, (c) *Tectocoris diophthalmus*, (d) *Coccinella transversalis*, (e) *Trithemis aurora*, (f) *Taxila haquinus*. Photo © MK Khan.

Insects exhibit species‐specific, population‐specific, and sex‐specific body colours and patterns, which can also vary across life stages (Figure 1) (Khan, 2020; Khan & Herberstein, 2020b; Wittkopp & Beldade, 2009). Insect colour originates from the pigments that are deposited underneath the cuticle, from cuticular surface structures, or a combination of both (Chapman & Chapman, 1998). These colours may function in interspecific communication (e.g. aposematism, crypsis including mimicry and camouflage), intraspecific communication (e.g. signalling), thermoregulation, and UV protection (Caro, 2005; Cott, 1940; Figon & Casas, 2018; Futahashi, 2020). Often, colour is associated with multiple functions simultaneously, and sometimes in conflicting ways. For example, the non‐territorial damselfly *Xanthagrion erythroneurum* undergoes ontogenetic colour change from yellow to red shortly after emergence, which signals sexual maturity but may also increase predation risk (Khan & Herberstein, 2020a). Similarly, the larger aposematic orange patches on the black body of moth larvae are effective in predator deterrence but are less effective in thermoregulation (Lindstedt et al., 2009). Appreciating the complexity of body colours and their function is of utmost important in understanding the local adaptation and evolution of populations (Endler & Mappes, 2017).

Insects can respond to anthropogenic climate change through the plastic responses of individuals (Buckley et al., 2017; Hodgson et al., 2011) or through genetic and microevolutionary changes in populations (Larson et al., 2019; Overgaard et al., 2014; Ranga et al., 2017). There are several lines of evidence (temporal, geographical, and experimental studies) that indicate that insect colours vary with climate such as temperature and humidity (Lis et al., 2020; MacLean et al., 2019; Wilts et al., 2019; Xing et al., 2018). For example, Zvereva et al. (2019) observed a reduction of the darker subarctic leaf beetle morphs (*Chrysomela lapponica*) in conjunction with an increase of spring temperatures by 2.5°C. However, the relationship between climate and insect colour is complex with several biotic and abiotic factors at play (reviewed in Clusella‐Trullas & Nielsen, 2020). For example, adaptive change to new climatic conditions can increase survivability, but can also carry reproductive fitness costs (Candolin & Heuschele, 2008). Understanding the capacity of insects to respond to a changing climate is important for estimating the extinction risk of populations and species (Halsch et al., 2021; True, 2003; Urban, 2015).

The aim of this review is to examine the contemporary evidence of insect responses (colour change) against a rapidly changing climate (Table 1) and review the impact of climate‐driven colour change on intraspecific communication in insects (Table 2). We highlighted the current gaps and proposed future directions where further research is required. We believe, our review will provide insights into how insect colour varies across climates and will highlight the ecological and evolutionary consequences of such variation. We recognise that colour is a multi‐component phenomenon that integrates overall intensity (the human‐based perception of the overall intensity of light emitted or reflected from a stimulus) and quality (i.e., the perceived category of colour, determined by the spectral shape of a stimulus; see definitions by Kemp et al., 2015). For the purpose of this review, we mostly consider the perceived intensity of light reflected from the insect body and use the term ‘lightness’ as this is frequently used in the literature we reviewed.

**TABLE 1:** Evidence of insect colour change associated with latitude and climatic factors. Study type refers to whether the study used temporal, geographic, or experimental evidence of colour change.

Species	Order	Study type	Insects' response	Factors associated with colour change	References
*Colias meadii*	Lepidoptera	Temporal	Decreased wing melanisation	Warmer temperature	MacLean et al. (2016)
*Colias meadii*	Lepidoptera	Temporal	Increased wing melanisation	Higher temperature	MacLean et al. (2019)
Butterflies and dragonflies	Odonata	Temporal	Decreased melanisation	Higher temperature	Zeuss et al. (2014)
*Adalia bipunctata*	Coleoptera	Temporal	Decreased frequency of melanic morph	Higher spring temperatures	Brakefield and de Jong (2011)
*Chrysomela lapponica*	Coleoptera	Temporal	Decreased darker morphs	Higher spring daily temperatures	Zvereva et al. (2019)
*Timema cristinae*	Phasmatodea	Temporal	Increased frequency of melanic morphs	Warmer temperature	Nosil et al. (2018)
*Adalia bipunctata*	Coleoptera	Geographical	Decreased frequency of melanic morphs	Altitude	Scali and Creed (1975)
*Oreina sulcate*	Coleoptera	Geographical	Green colours	Lower elevations	Mikhailov (2001)
*Oreina sulcate*	Coleoptera	Geographical	Darker and more reflective metallic morphs	Higher elevations	Mikhailov (2001)
*Colias eurytheme*	Lepidoptera	Geographical	Darker hindwing (undersides)	Higher latitude	Watt (1968)
Bumblebees	Hymenoptera	Geographical	Darker colour	Lower latitude	Williams (2007)
*Drosophila melanogaster*	Diptera	Experimental	Decreased colour on the thorax and abdomen	Higher temperature	Gibert et al. (1998)
*Saccharosydne procerus*	Hemiptera	Experimental	Darker colour	Higher temperature	Yin et al. (2015)
*Pachydiplax longipennis*	Odonata	Experimental	Increased wing ornamentation	Warmer larval temperatures	Lis et al. (2020)
*Danaus plexippus*	Lepidoptera	Experimental	Greater portion of black and a lower portion of white and yellow colour	Lower temperature	Solensky and Larkin (2003)

**TABLE 2:** Impact of climate‐driven colour change on sexual selection.

Species	Order	Factors associated with colour change	Impact	References
*Phymata americana*	Hemiptera	Temperature	Dark individuals had a higher success rate in mate searching at colder ambient temperature	Punzalan et al. (2008)
*Pachydiplax longipennis*	Odonata	Temperature	Greater abundance of dark pigment in the wing increased male flight performance at colder temperature	Moore et al. (2019)
*Ischnura elegans*	Odonata	High latitude	Darker colours led to increased sexual conflict	Svensson, Willink, et al. (2020)
*Allonemobius socius*	Orthoptera	Short season length	Darker colours led to increased melanin‐based immunity	Fedorka et al. (2013)
*Colias philodice eriphyle*	Lepidoptera	Elevation	Lighter males had reduced flight activity at high elevation	Ellers and Boggs (2004)

## 2 INSECT COLOUR: COLOUR PRODUCTION MECHANISM AND LINK TO ENVIRONMENTAL FACTORS

Insect colour is involved in mimicry, camouflage, thermoregulation, and intraspecific communication (Cott, 1940; Khan & Herberstein, 2021; True, 2003). In addition to colour production, the pigment melanin is also involved in immunological protection against pathogens and parasites (Armitage & Siva‐Jothy, 2005; Mackintosh, 2001; Wilson et al., 2001) as a rate‐limiting molecule of the phenoloxidase cascade (José de Souza et al., 2011; Sugumaran & Barek, 2016). For more details on the function of insect colours, we refer the reader to several excellent reviews (Badejo et al., 2020; Figon & Casas, 2018; True, 2003; Umbers et al., 2014).

The colours of insects are mostly generated by pigments and/or structures. Pigments or their precursor can either be synthesised in epidermal cells or extracted from diet (e.g. carotenoids) (Dresp, 2014; Wittkopp & Beldade, 2009). There are eight classes of pigments (melanins, ommochromes, pteridines, tetrapyrroles, carotenoids, flavonoids, papiliochromes, and quinones) that are involved in insect colouration (Futahashi & Osanai‐Futahashi, 2021). Of these, melanins, ommochromes, and pteridines are the dominant colour pigments in dragonflies (Futahashi & Osanai‐Futahashi, 2021), while tetrapyrroles, carotenoids, flavonoids, papiliochromes, and quinones are the main contributors to colour in grasshoppers (Futahashi & Osanai‐Futahashi, 2021), aphids (Tsuchida, 2016), and butterflies (Burghardt et al., 2000; Stavenga et al., 2014b). Pigments can also contribute to structural colours in insects (Yoshioka & Kinoshita, 2006).

Structural colours are the result of light refraction, interference, or diffraction caused by photonic structures in the insect integument (Kemp et al., 2006; Stuart‐Fox et al., 2017; Sun et al., 2013; Vukusic & Sambles, 2003). Structural colours are common in butterflies (Burg & Parnell, 2018; Stavenga et al., 2014a), moths (Stavenga et al., 2018) and beetles (Burg & Parnell, 2018), and Saharan silver ants (reviewed in Stuart‐Fox et al., 2017). For example, the metallic colours of beetles are generated by epicuticular multilayer reflectors (McNamara et al., 2012). In addition to pigmentation and structural colour, some insects, such as fireflies, beetles, and springtails also produce colour by luciferases, an enzyme capable of producing light in bioluminescence (Viviani, 2002) a form of colour production that we will not consider further in this review.

The expression of insect colours in terms of quantity and quality can be impacted by environmental factors including temperature, rainfall, and solar radiation (Cott, 1940; Elith et al., 2010). Temperature directly affects pigment production (Hassall & Thompson, 2012), and insects in colder environments tend to be darker, as melanin production is greater in lower temperatures (De Souza et al., 2017). The selective advantage of this response to environmental temperature is that darker bodies can absorb more solar radiation than lighter bodies, allowing darker individuals to remain active for longer periods in colder regions, which may increase reproductive success and feeding rates (Clusella Trullas et al., 2007; De Souza et al., 2017). In support of this idea, male damselflies (*Calopteryx splendens*) with high pigmentation and larger wing spots had significantly faster early morning activation than males with smaller wing spots, presumably because the larger dark patches warmed up the insect bodies faster (Laakso et al., 2021). A related but distinct proposed function of greater melanin pigmentation is the absorption of damaging UV radiation. For example, pierid butterflies under high levels of solar radiation produced more melanin (Stelbrink et al., 2019). Finally, geometrid moths became increasingly brighter with increasing solar radiation along a latitudinal gradient but not an altitudinal gradient, where the moths became darker (Heidrich et al., 2018). The lack of consistent patterns with temperature and irradiation may be an indicator of species or population‐specific trade‐offs between UV protection and thermoregulation because the intensity of UV radiation and temperature are positively associated – hotter environments also have higher UV radiation, which may lead to trade‐offs in melanin‐related functions (see Clusella‐Trullas & Nielsen, 2020).

Humidity can also trigger colour change in insects, even within the same individual, such as in *Adscita statice*, a green forester moth that changes its colour at dusk and dawn with humidity (Wilts et al., 2019). The ambient humidity modifies the multilayer refractive index which alters the moth's colour from red to green (Wilts et al., 2019). Moreover, male Hercules beetles, *Dynastes hercules*, switch the colour of the elytra from black (at night) to yellow (in the morning) associated with a humidity shift from high to low (Hinton & Jarman, 1973). There is conflicting evidence that insect melanisation increases with decreasing humidity thereby reducing cuticular water loss and increasing resistant to desiccation – the melanism–desiccation hypothesis. Law et al. (2020) found indirect support where ant assemblages in the canopy experiencing dryer conditions were darker than assemblages on the more humid ground. However, results from an experiment that selected for darker and lighter phenotypes of *Drosophila melanogaster* over generations found no relationship between desiccation tolerance and colour (Rajpurohit et al., 2016). It is possible that there are other physiological mechanisms that are responsible for desiccation tolerance in insects. As might be expected, the response of organisms to environmental change is complex, highly context‐dependent and is shaped by both their physical and biological environments.

## 3 EVIDENCE OF CLIMATE CHANGE IMPACT ON INSECT COLOUR

### 3.1 Temporal studies

Colour change in insect over time has been linked to climate change. A long‐term study (1953–2012) on *Colias meadii* butterflies in the USA showed that wing melanisation decreased with increasing temperature during this time period (MacLean et al., 2016). This pattern, however, is not true across space. In the same species, over the same time period, melanism decreased with increasing temperature in Northern Canada but increased with increasing temperature in southern USA (MacLean et al., 2019). Explaining the mechanisms that drive these seemingly disparate patterns is challenging, and to date no unifying explanation has emerged (MacLean et al., 2019). Different patterns of insect colour responses to increasing temperature are also apparent in other taxa. For example, European dragonfly individuals are lighter in colour in warmer regions (Zeuss et al., 2014) and the melanic morphs of two‐spot ladybird beetle, *Adalia bipunctata*, (Brakefield & de Jong, 2011) and leaf beetles (*Chrysomela lapponica*) have decreased with an increase of spring temperatures over the last 25 years (Zvereva et al., 2019). This may be due to the loss of thermoregulatory advantages of darker individuals in warmer springs. Conversely, the frequency of melanic stick insects (*Timea cristine*) morphs increased in warmer years because of darker individuals have a crypsis advantage on dry and brownish plants in warmer years (Nosil et al., 2018).

### 3.2 Geographic variation

Phenotypic differences across latitude and/or altitude are often used to anticipate how organisms might react to climate change (Fielding et al., 1999; Golab et al., 2022). Altitude (or elevation) is linked to colour pattern polymorphism in several insect species (Hodkinson, 2005) whereby, the frequency of melanic morphs increases with altitude (Berry & Willmer, 1986; Hodkinson, 2005). Species, such as spittlebugs *Philaenus spumarius* (Berry & Willmer, 1986), dung beetles *Onthophagus proteus* (Stanbrook et al., 2021), *Eupteryx* leafhoppers (Stewart, 1986), grasshoppers (Guerrucci & Voisin, 1988), and noctuid moths (Heidrich et al., 2021) showed increased melanisation with altitude. However, in some ladybird beetles (*Adalia bipunctata*) (Scali & Creed, 1975) and bush cricket (*Isophya rizeensis*) (Kuyucu et al., 2018) the melanic frequencies decreased with altitude. Similarly, in geometrid moths in China, the observation of darker colour moths at higher elevations was not consistent across different study sites (Xing et al., 2018). In addition to melanisms, structural colours that cause a metallic appearance also change with elevation. For example, the metallic colouration in *Oreina sulcata* beetle varied with elevation: green‐colour morphs were more frequent at lower elevations, and darker and more reflective metallic morphs at higher elevations (Mikhailov, 2001).

Distributions across different latitudes can also relate to colour variation in insects (Raffard et al., 2020; Zheng et al., 2015). A study conducted on monarch caterpillars (*Danaus plexippus*) over a 650,000‐km^2^ area in the USA and Canada showed comparatively less pigmentation at lower latitude or warmer locations than in individuals found at higher latitude or colder locations (Tseng et al., 2023). Rather than a continuous effect, latitude can also have a bimodal effect: individuals tend to be darker both at higher latitude (i.e. in colder climates) and lower latitude (in warmer climate), with lighter morph at intermediate latitudes (Stewart, 1986; Watt, 1968; Williams, 2007). For example, *Colias* butterflies possess darker hindwing undersides at higher latitude and colder climates as well as lower latitudes and hotter climates (Watt, 1968).

By contrast, some insects are generally darker in colder climates and lighter in warmer climates (Bishop et al., 2016; Pinkert et al., 2018; Zeuss et al., 2014). For example, *Tectocoris diophthalmus* bugs at temperate and lower latitude sites showed larger patches of blue against a lighter red background compared with subtropical and tropical bugs (Fabricant et al., 2018). On the other hand, in adult swallowtail butterflies (*Sericinus montelus*), males at lower latitudes were more likely to express darker colours than males at higher latitudes (Zheng et al., 2015). Similar results were also found in bumblebees (Williams, 2007). Some of the observed contradictions described above may reflect that habitats and climate variables are interwoven and complex, requiring controlled experiments to isolate the effect of temperature on colour.

## 4 EXPERIMENTAL EVIDENCE OF TEMPERATURE IMPACT ON INSECT COLOUR

Several experimental studies investigated how temperature affects insect colour. In *Drosophila melanogaster*, pigmentation on the thorax and abdomen decreased with experimentally increased temperature (Gibert et al., 1998). Contrary to this result, planthoppers *Saccharosydne procerus* produced darker colours when reared at higher temperatures (Yin et al., 2015). Similarly, male territorial dragonflies, *Pachydiplax longipennis*, produced darker wing ornamentation when larvae were reared at higher temperature than when larvae were reared at lower temperature (Lis et al., 2020). A controlled rearing experiment in harlequin bug (male and female *Murgantia histrionica*) also showed that temperature was a significant factor for melanisation: individuals reared at lower temperature were darker than the individuals at higher temperature (Sibilia et al., 2018). Similarly, the wood tiger moth (*Arctia plantaginis*) reared at higher temperatures was less pigmented than individuals reared at lower temperatures (Galarza et al., 2019). Finally, monarch larvae (*Danaus plexippus*) reared at lower temperatures developed a higher portion of black and a lower portion of white and yellow, compared with larvae reared at warmer temperature (Solensky & Larkin, 2003). Overall, while it is clear from these studies that temperature influences insect colour expression, patterns between experiments and species are quite variable.

Some of the responses to rearing temperature can result in seasonal polymorphism: *Colias* butterflies, *Papilio machaonin*, and *Pontia* butterflies show seasonally polyphenic traits that can generate various adaptive phenotypes in response to seasonal environmental variation (Kingsolver, 1995). Distinct wing phenotypes are the most common seasonal polyphenism in butterflies that can influence their thermoregulatory ability (Kingsolver, 1987). For example, environmental manipulation such as altering photoperiodic conditions, which resulted in more hours at higher temperatures, during the larval stage of the white butterfly (*Pontia occidentalis*), lead to greater melanisation on the dorsal forewings and lower melanisation on the ventral hindwings of summer individuals compared with spring individuals (Kingsolver, 1995; Kingsolver & Wiernasz, 1991). Significantly, the different phenotypes had different survivability under extreme thermal conditions in the field (Kingsolver, 1995).

Some insects are also able to change colour reversibly with ambient temperature (Huang & Reinhard, 2012; Key & Day, 1954; O'Farrell., 1964; Umbers et al., 2013). In common blue‐tail damselflies (*Ischnura heterosticta*), morphs changed their colour partially and reversibly under controlled laboratory conditions: dull green or grey colour under 12°C and bright blue above 15°C (Huang & Reinhard, 2012; O'Farrell., 1964). Similarly, male chameleon grasshopper (*Kosciuscola tristis*) also showed rapid reversible colour change under different laboratory conditions‐ black at 10°C, intermediate colouration from 10 to 15°C and turquoise colouration over 25°C (Key & Day, 1954; Umbers, 2011; Umbers et al., 2013, 2014).

## 5 MECHANISMS: PHENOTYPIC PLASTICITY, MICROEVOLUTIONARY RESPONSE

Populations experiencing new selection pressures may respond in three different ways – they may shift to a more suitable habitat, adjust to changing conditions through phenotypic plasticity, or they may adapt to new conditions through population genetic change (Davis et al., 2005; Holt, 1990). Generally, plastic responses to new conditions are more rapid than evolutionary responses (Sgrò et al., 2016). The precise mechanism depends on life history traits, dispersal ability, availability of alternative habitats, and the rate of continual environmental change (Gienapp et al., 2008). Sometimes populations combine all three possible responses to climatic change (Davis & Shaw, 2001).

In insects, phenotypic plasticity of colour expression can stem from a change in the colour pigment in the epidermis or the cuticle (Nijhout, 2010). For instance, RNA interference (RNAi) treatment of yellow mealworm (*Tenebrio molitor*) showed light brownish colour whereas, enzymes deficient in the cuticle tanning pathway resulted in darker pigments (Mun et al., 2020). Similarly, swallowtail butterfly (*Papilio xuthus*) displayed black cuticle colour when epidermal cells expressed tyrosine hydroxylase and dopa decarboxylase enzymes whereas they exhibited reddish‐brown colour during the epidermal expression of tyrosine hydroxylase, dopa decarboxylase, and ebony enzymes (Futahashi & Fujiwara, 2005). Phenotypic variation of colour across seasons, that is, polyphenism (Nijhout, 2010) is known in many insects such as moths (*Orgyia antiqua*) (Sandre et al., 2007), narrow‐headed ants (*Formica exsecta*) (Putyatina et al., 2022) and butterflies (species belong to tribe Junoniini) (Clarke, 2017).

Phenotypic plasticity provides an important mechanism to adjust to new environmental conditions. The underlying mechanisms are likely to be the up and downregulation of the relevant genes. In *Colias crocea* butterflies, an increased expression of the BarH‐1 gene is responsible for the white wing colour (Woronik et al., 2019). In *Heliconius* butterflies *optix* and *cortex* genes control red and yellow/white wing patterns (Jiggins et al., 2017). Furthermore, in *Ischnura senegalensis* damselflies, the expression of *ebony* and *black* genes is responsible for the reddish‐brown colour in the thorax of the gynochrome female (Takahashi et al., 2019). The expression of colour producing genes may vary in response to climate change, however, experimental evidence for changing gene expressions is limited mostly because of the nature and complexity of the genetic basis for colour (Clusella‐Trullas & Nielsen, 2020; Daniels et al., 2014; Roulin, 2014). Recent advancement in genetics and genomics now provide platforms to study the mechanisms of insect colour change in response to climate.

It has been argued that phenotypic plasticity, as described above, is unable to provide long‐term solutions for populations (Gienapp et al., 2008; Przybylo et al., 2000). Hence, microevolutionary responses are required to cope with continual environmental change over long periods (Davis et al., 2005; Stockwell et al., 2003). While the heritability of melanism is thought to be high (Roff & Fairbairn, 2013), potentially setting the stage for rapid evolution, insect melanin is associated with several other physiological mechanisms, such as immunity and desiccation, which could potentially counteract adaptive colour evolution in response to a warming climate (Clusella‐Trullas & Nielsen, 2020). In the next section, we specifically discuss how climate‐induced colour change might interact with sexual signals in insects.

## 6 SEXUAL SELECTION AND MODE OF ACTION

Sexual selection is an important selective force that drives the evolution of pre‐ and post‐copulatory traits (Durrant et al., 2020; Hosken & House, 2011) and can ultimately accelerate speciation (Hugall & Stuart‐Fox, 2012). Colour in a sexual context is mostly associated with visual signalling that may facilitate mate recognition and communication of mate or competitor quality (Khan, 2020; Khan & Herberstein, 2021). Less obvious is the interaction of sexual colour signals and thermoregulation. While darker colours are frequently favoured by sexual selection, they also absorb more solar radiation and increase body temperature (Leith et al., 2022). A higher body temperature can be advantageous if it allows individuals to actively search for mates. For example, darker male speckled wood butterflies, heat up faster and are more likely to patrol for females than lighter males that perch (Van Dyck & Matthysen, 1998). Presumably, increased melanisation driven by sexual selection could be detrimental in warmer or hotter climates, however, we could not find research to confirm this prediction.

### 6.1 Intrasexual selection

Intrasexual selection involves individuals of one sex (usually, but not exclusively, males) that compete with individuals of the same sex over access to mates. Mate competition can take the form of fighting and defending territories and/or resources selecting traits that facilitate fights and defence (Fitze et al., 2008). Alternatively, scrambling does not involve intrasexual interactions, selecting traits that facilitate fast emergence, location, and approach of mates (see Herberstein et al., 2017 for a review). Colour may also be a trait selected for intrasexual competition, such as in the damselfly *Calopteryx haemorrhoidalis*, where males with higher wing melanisation have a greater ability to successfully defend territories (Córdoba‐Aguilar, 2002). These more conspicuous males have an advantage by outcompeting rivals and attracting more mates. In non‐territorial mating system conspicuous colour can evolve to reduce unprofitable matting, especially in species where population density and competition for mating is high. For example, conspicuous blue abdominal stripes in *Xanthagrion erythroneurum* male damselflies signal unprofitabilty as mate and thus reduce male–male mating interactions in high male density populations (Khan & Herberstein, 2019).

### 6.2 Intersexual selection

Intersexual selection occurs when members of one sex select a mate based on their physical appearance or traits. This form of selection is common in females and males invest in physical traits and their display (Fitze et al., 2008) or in providing direct benefits such as nutritional resources (Møller & Jennions, 2001). There are many examples where females choose males with exaggerated traits such as conspicuous colour or elaborate ornamentation (Andersson, 1994; Darwin, 1888). In *Colias philodice eriphyle*, butterflies male wing colour is attractive to females – more melanised males are preferentially chosen by females (Ellers & Boggs, 2003). Colour can also signal an unprofitable mate and warn off unwanted mate. For example, sexually immature *Agriocnmis femina* females signal their unprofitability as mates with conspicuous red colour which reduces male mating attempts with pre‐reproductive females (Khan, 2020). For both modes of sexual selection, climate‐driven colour change may impair signalling and activity patterns, courtship displays, and ultimately reproductive fitness.

## 7 IMPACT OF CLIMATE‐DRIVEN COLOUR CHANGE ON SEXUAL SELECTION

Climate change may impact life history traits and mating systems that subsequently affect the strength or direction of sexual selection (Maan & Seehausen, 2011; Pilakouta & Ålund, 2021). A recent quantitative genetic model showed that the strength of sexual selection may decrease due to rapid climate change, which reduces the benefits of sexual selection relative to the survival benefits of adapting to new environmental conditions (Martinossi‐Allibert et al., 2019). For example, temperature can determine the outcome of sexual selection by changing reproductive behaviour, such as mate searching, male–female, and male–male interactions (García‐Roa et al., 2020). Similarly, a study conducted on ambush bugs, *Phymata americana*, showed that the interaction between ambient temperature and melanisation could affect the outcome of mate competition as male bugs with relatively darker colour patterns had higher mate‐searching success in cool ambient temperatures but may suffer detrimental effects in warm ambient temperature (Punzalan et al., 2008).

Physiologically, a warming climate may enhance the fitness of animals living in cooler temperature and higher latitudes whereas increasing temperature is likely to have detrimental consequences on tropical animals (Deutsch et al., 2008). Behaviourally, animals that display sex‐specific traits in an inter or intrasexual context may also be affected by increasing temperature (Moore et al., 2019). On the one hand, higher temperatures may increase mating opportunity and reproductive output, however, more conspicuous sexual signals or signalling may incur costs if they are more likely to be detected by parasites and predators (Halfwerk et al., 2011; Patricelli & Blickley, 2006; Zuk et al., 2006). In addition, melanised wing interference patterns or patches in *Drosophila* or dragonflies might increase reproductive success but may be physiologically detrimental as they increase body temperature in warmer climates (Corbet, 1999; Katayama et al., 2014; Moore et al., 2021). Hence, selection may reduce sexual colour signals at higher temperatures to mitigate costs, as shown in some dragonflies. Male dragonflies with higher wing melanisation had greater mating success than males with less melanised wings (Moore et al., 2021). However, wing melanisation also increased individual body temperature by >2°C (Moore et al., 2019; Svensson, Gomez‐Llano, & Waller, 2020; Svensson & Waller, 2013). Such thermal effects may confer modest locomotor benefits in low‐temperature environments but may reduce flight ability, damage wing tissue, and cause death in high temperature environments (Moore et al., 2019; Svensson, Gomez‐Llano, & Waller, 2020). This impact may be sex‐specific as females forage at lower temperatures or in shaded micro‐habitats (Moore et al., 2021).

The above examples represent isolated case studies in the absence of a predictive framework on how insects might respond to the climate change impacts on sexual signals and sexual selection. Below we attempt to generate some broad non‐mutually exclusive predictions that we hope will serve for future direct and indirect testing. (1) Reduced melanisation in warmer but lower altitude areas is likely to be associated with a shift in signal preference toward lighter colour signals; (2) at higher altitude melanisation will be less affected due to the additional function of UV protection and signal preference for darker signals will be maintained; (3) courtship displays in species with darker sexual signals will shift in season and daytime toward earlier (cooler) seasons and earlier (cooler) daytime hours.

## 8 KNOWLEDGE GAPS AND FUTURE DIRECTIONS

Our review identified indirect and direct evidence that climate, specifically temperature, affects colour expression, in particular melanin‐based colours in insects. Many studies we reviewed provided indirect evidence using time series or spatial patterns, while experimental studies were less frequent. Perhaps not surprisingly, experimental studies tended to use ‘model’ species such as *Drosophila* (Ramniwas & Singh, 2022) or *Colias* butterflies (Nielsen & Kingsolver, 2020) with fewer examples from non‐model species. This raises the question of whether the model‐species responses can be extrapolated to other species or taxonomic groups (Zuk et al., 2014). Somewhat surprising was the inconsistency in response between and sometimes within species: higher temperatures led to both increases and decreases in melanisation or the frequency of melanic phenotypes, with no clear pattern emerging. Regardless of the direction of response, fitness consequences of climate change‐induced colour change in terms of reproduction, foraging, and survival were mostly not explicitly tested but for some outstanding examples (e.g. butterflies (Xing et al., 2016; Zeuss et al., 2014); fig wasps (Jevanandam et al., 2013); ladybird beetle (Dubey et al., 2016)).

We can see great opportunities in balancing short‐term experiments that are most likely to detect phenotypic plasticity with long‐term experiments over several generations that are likely to capture microevolutionary effects to generate a more wholistic assessment of how insect populations might be affected by climate change. We also observed possible geographic and sex‐specific biases in the current literatures due to the limited geographic regions (mostly the temperate regions of the Northern Hemisphere) and a focus on male colour signals. Clearly, large‐scale geographic surveys on both sexes of multiple species can reduce this bias. The availability of many advanced techniques such as digital photographs for assessing colour, and computer‐assisted image analysis software also opens the use of museum specimen that may be too fragile for conventional photospectrometry. Usage of museum specimens provides further opportunity to understand the temporal trend of insect colour change under a changing climate. The advancement of genomics, bioinformatics, and genetics also broaden the scope to understand the genetic mechanism of climate change‐induced colour change. In conclusion, the effect of global climate change on insect colour can impact physiological functions, intra‐ and interspecies communication, and sexual selection, all of which may contribute to the global decline of insects. We believe monitoring the impact of global climate change on insect traits will assist the management of biodiversity and environmental sustainability.

We further highlighted the following questions to improve our understanding of climate change impact on insect colour, sexual signals, and sexual selection:

1. How does global warming drive insect colour change in different populations, species, and sexes?
2. How do temperature, humidity, life history traits, sexual selection, or a combination thereof relate to variation in colour under climate change?
3. How does climate‐related colour change impact mechanism of sexual selection such as intrasexual competition (male–male competition, female–female competition) and intrasexual mate preferences (male mate choice or female mate choice)?
4. How does climate‐related colour change and its impact on sexual signals and sexual selection vary across biomes; do tropical and temporal species respond similarly or differently?
5. How does climate‐related colour change impact species–species interactions such as predator–prey and host–pathogen interactions?
6. How does colour change and sexual signalling under a rapidly changing climate impact insect fitness?

## STATEMENT OF DIVERSITY AND INCLUSION

We strongly support equity, diversity, and inclusion in science (Rößler et al., 2020). The authors come from different countries (Bangladesh, Austria, and Australia) and represent different career stages (Masters student, Early career researcher, & Professor). One or more of the authors self‐identifies as a member of the LGBTQI+ community. One or more authors are from an underrepresented ethnic minority in science.

## AUTHOR CONTRIBUTIONS

**Md Tangigul Haque:** Conceptualization (equal); writing – original draft (lead). **Md Kawsar Khan:** Conceptualization (equal); writing – review and editing (lead). **Marie E. Herberstein:** Conceptualization (equal); writing – review and editing (lead).

## FUNDING INFORMATION

TH was supported by International Macquarie University Research excellence scholarship (iMQRES).

## CONFLICT OF INTEREST STATEMENT

The authors declare no competing interests.

## Data Availability

Data sharing not applicable to this article as no datasets were generated or analysed during the current study.
